# Pain phenotyping and investigation of outcomes in physical therapy: An exploratory study in patients with low back pain

**DOI:** 10.1371/journal.pone.0281517

**Published:** 2023-02-14

**Authors:** Abigail T. Wilson, Joseph L. Riley, Mark D. Bishop, Jason M. Beneciuk, Yenisel Cruz-Almeida, Keri Markut, Charlotte Redd, Nicholas LeBlond, Patrick H. Pham, David Shirey, Joel E. Bialosky

**Affiliations:** 1 School of Kinesiology and Rehabilitation Sciences, College of Health Professions and Sciences, University of Central Florida, Orlando, Florida, United States of America; 2 Department of Community Dentistry and Behavioral Science, University of Florida, Gainesville, Florida, United States of America; 3 Pain Research & Intervention Center of Excellence, University of Florida, Gainesville, Florida, United States of America; 4 University of Florida Department of Physical Therapy, Gainesville, Florida, United States of America; 5 Clinical Research Center, Brooks Rehabilitation, Jacksonville, Florida, United States of America; 6 University of Florida Health Rehab Center-Orthopedic and Sports Medicine Institute, Gainesville, Florida, United States of America; 7 Duke University Health System Durham, North Carolina, United States of America; 8 Brooks Rehabilitation, Jacksonville, Florida, United States of America; Mugla Sitki Kocman Universitesi, TURKEY

## Abstract

Phenotypes have been proposed as a method of characterizing subgroups based on biopsychosocial factors to identify responders to analgesic treatments. This study aimed to, first, confirm phenotypes in patients with low back pain receiving physical therapy based on an *a priori* set of factors used to derive subgroups in other pain populations. Second, an exploratory analysis examined if phenotypes differentiated pain and disability outcomes at four weeks of physical therapy. Fifty-five participants completed psychological questionnaires and pressure pain threshold (PPT). Somatization, anxiety, and depression domains of the Symptom-Checklist-90-Revised, and PPT, were entered into a hierarchical agglomerative cluster analysis with Ward’s method to identify phenotypes. Repeated measures ANOVAs assessed pain ratings and disability by phenotype at four weeks. Three clusters emerged: 1) high emotional distress and pain sensitivity (n = 10), 2) low emotional distress (n = 34), 3) low pain sensitivity (n = 11). As an exploratory study, clusters did not differentiate pain ratings or disability after four weeks of physical therapy (p’s>0.05). However, trends were observed as magnitude of change for pain varied by phenotype. This supports the characterization of homogenous subgroups based on a protocol conducted in the clinical setting with varying effect sizes noted by phenotype for short-term changes in pain. As an exploratory study, future studies should aim to repeat this trial in a larger sample of patients.

## Introduction

Globally, the need for rehabilitation services is large with 2.41 billion individuals having a neuromusculoskeletal condition that would benefit from a non-pharmacologic treatment approach [1]. Low back pain (LBP) is the most prevalent condition contributing to this need[1] and the leading cause of years lived with disability [2]. A current challenge in the management of LBP is the large variation in rehabilitation outcomes [3,4], which may stem from a discrepancy between the heterogenous nature of LBP and a traditional ‘one size fits all’ approach that is commonly applied. Given the large need for rehabilitation of LBP [1] and inconsistent outcomes, a more precise approach to inform clinical decision making is needed.

Treatment stratification identifies homogenous subgroups to optimize clinical outcome prediction and has been endorsed as a step toward precision medicine. However, current approaches have limitations. For example, clinical prediction rules fail to be replicated in validation studies [5,6]. Prognostic based approaches that incorporate the STaRT Back Tool show promising results [7–9]; however, recent results found no effect on the transition to chronic pain [10]. Additionally, these approaches focus on negative pain related psychological factors and symptom location yet do not account for other potentially relevant prognostic factors, such as pain sensitivity.

Phenotyping is a more comprehensive method of identifying homogenous subgroups based on the interaction between prognostic indicators [11], psychological [12], or pain sensitivity factors [13]. Phenotypes have the potential to differentiate pain trajectories and clinical outcomes [11]. In a study of patients receiving physical therapy for musculoskeletal pain, five phenotypes were identified based on eleven pain-related prognostic factors [14]. Greater variability in pain, function, and recovery [15] were observed at fifty-two weeks across phenotypes compared to the anatomical location of pain, suggesting phenotypes more precisely distinguished long-term trajectories [14]. Numerous studies have identified phenotypes; however, few have prospectively examined their utility in distinguishing short-term clinical outcomes in rehabilitation despite this being a research priority [11]. An additional limitation of the prior work is phenotypes were derived based on a number of clinical factors that may not be feasible to implement in the clinical setting. Phenotyping can also be challenging to implement in the clinical setting due to extensive clinician and patient burden.

In the Orofacial Pain Prospective Evaluation and Risk Assessment (OPPERA) study, reliable phenotypes of patients with temporomandibular dysfunction and healthy controls were derived based on somatization, depression, anxiety dimensions of the Symptom Checklist-90-Revised (SCL-90-R) as well as Pressure Pain Threshold (PPT) applied to the upper trapezius [16,17]. Three phenotypes were identified that distinguished individuals who developed chronic pain [16] and were further characterized by differences in sex, age, pain ratings, psychological factors, and quantitative sensory testing [17]. In addition to being validated in a large sample of individuals with mixed chronic pain conditions [17], this protocol could be feasibly implemented in a clinical setting due to the relatively low clinician and patient burden (one questionnaire and PPT). The prior studies examined this set of variables in patients receiving treatment at a multidisciplinary chronic pain clinic [17]; however, this had never been specifically applied to patients receiving physical therapy for LBP.

Therefore, this current research aimed to, first, identify if these phenotypes are present in patients receiving physical therapy for LBP based on the set of established [16] and validated [17] variables using clinically feasible measures. Second, as an exploratory analysis, this study prospectively examined if identified phenotypes were associated with differences in clinical outcomes of pain intensity and disability after 4 weeks of physical therapy. Four weeks were selected due to prior research selecting this time point for short-term outcomes [18,19]. We add to the existing body of literature by applying this approach to a new sample, patients receiving physical therapy, and examining phenotypes’ prognostic utility for rehabilitation outcomes.

## Materials and methods

Data for this observational, prospective cohort study was collected between December 2020-August 2021 from six outpatient physical therapy clinics within Brooks Rehabilitation in Jacksonville, FL, two outpatient physical therapy clinics within University of Florida Health in Gainesville, FL, and one outpatient physical therapy clinic within University of Florida Health in Jacksonville, FL. Thirteen physical therapists were recruited by word of mouth and underwent IRB-01 training and a 30-minute training session by the study coordinator to standardize data collection and recruitment methods. This study was approved by the University of Florida Institutional Review Board for Human Subjects Research. All participants provided written informed consent to enroll in the study.

### Participants

Consecutive participants were recruited by the patient’s physical therapist at the initial evaluation or first follow-up appointment using a standard script and study flyer. Participants between 18–75 years old who were currently receiving outpatient physical therapy for LBP at one of the approved study sites were eligible to participate. LBP was defined as pain between the inferior posterior margin of the ribs and the horizontal gluteal fold [20]. Participants who did not speak English, had a systemic medical condition known to affect sensation, or low back surgery or fracture within the past 6 months were excluded.

### Study overview

At baseline, enrolled participants completed self-reported demographic and psychological measures online using Research Electronic Data Capture (REDCap) tools, a secure online software designed for collecting data in research studies, hosted at the University of Florida. PPT was assessed at baseline and once weekly for four weeks during scheduled physical therapy sessions by physical therapists trained by the study coordinator. Baseline measurement was within 2 weeks of the initial evaluation and defined as the appointment after the patient enrolled in the study. Clinical pain ratings and the Oswestry Disability Index were collected at baseline and 4 weeks, allowing for assessment of short-term clinical outcomes. Four weeks was selected due to prior literature selecting this as a measurement time point for short term outcomes.(7) Patients received treatment at the discretion of their physical therapist.

### Measures

#### Measures to build phenotypes

Based on results of the original phenotyping trial^16^ as well as a follow-up validation study [17], somatization, anxiety, depression, and PPT applied to the upper trapezius and lower back were selected as *a priori* clustering variables.

#### Symptom Checklist-90-Revised (SCL-90-R)

The SCL-90-R [21,22] is a valid 90-item questionnaire in which individual items are scored from 0 (not at all) to 4 (extremely) and higher scores indicate greater psychological distress. The SCL-90-R measures 9 psychological domains: somatization, anxiety, depression, obsessive-compulsive, interpersonal sensitivity, hostility, phobic anxiety, paranoid ideation, psychoticism. Only the somatization, anxiety, and depression subscales were included in the cluster analysis. The SCL-90R has been previously applied to patients with low back pain [23–25].

#### Pressure-Pain Threshold (PPT)

A digital pressure algometer (Wagner Instruments FPX 25, Greenwich, CT) with a 1 cm diameter rubber tip was applied at 1 kgf/s to two locations: 1) locally at the low back medial to the posterior superior iliac spine on the most painful side and 2) remotely to the upper trapezius ipsilateral to the most painful side of LBP. Participants positioned in prone for the low back site and in sitting for the upper trapezius site. Participants were instructed to indicate when the sensation first changed from pressure to pain (pain threshold). This procedure was repeated two times [26] and the average PPT analyzed. PPT demonstrates excellent intra-rater reliability and good to excellent inter-rater reliability in patients with low back pain [27,28].

#### Measures used to characterize phenotypes

Study participants completed a demographic and clinical form including: gender, age, race, ethnicity, employment status, marital status, educational level, pain duration.

#### Pain Catastrophizing Scale (PCS)

The PCS is a 13-item valid [29–31] questionnaire in which individuals respond to a statement on a scale from 0 to 4. Scores range from 0–52 with higher scores indicating higher catastrophizing levels.

#### Fear-Avoidance Beliefs Questionnaire (FABQ)

The FABQ is a valid [32–34] measure of fear avoidance in patients with LBP. Items are scored from 0–6 with higher scores indicating greater fear avoidance. The FABQ includes a 7-item work subscale (scores range from 0–42 points) and a 4-item physical activity subscale (scores range from 0–24 points).

#### Tampa Scale of Kinesiophobia (TSK)

The TSK is an 11-item questionnaire with acceptable psychometric properties [35] that quantifies the fear of movement and injury/re-injury. Individual items are scored from 1 to 4 with totals ranging from 11–44. Higher TSK scores indicate greater kinesiophobia.

#### Pain Self-Efficacy Questionnaire (PSEQ)

The PSEQ measures the degree of pain related self-efficacy. The PSEQ consists of 10 items scored from 0–6 with totals ranging from 0–60. Higher scores indicate elevated levels of pain-related self-efficacy [36].

#### Pittsburgh Sleep Quality Index (PSQI)

The PSQI is a valid [37,38] self-report measure of sleep quality and disturbance in the past month consisting of 19 items with 7 components totaling a score ranging from 0–21 [39]. A higher score indicates a poorer sleep quality.

### Selection of measures used to determine short-term outcomes

#### Clinical pain ratings

Current, best, and worst clinical pain ratings over the past 24 hours were reported using a 101-point numeric pain rating scale (NRS) where 0 = no pain and 100 = worst pain imaginable [40–44]. Mean pain ratings were calculated by averaging the participant’s current, best, and worst LBP ratings.

#### Oswestry Disability Index (ODI)

The ODI is a 10-item valid [45] questionnaire that examines perceived disability specific to LBP. Participants answered questions on a 6-point scale with total scores ranging from 0–50 points (0–100%). Higher scores indicate greater perceived disability.

### Statistical analysis

We acknowledge that recommendations vary with some recent sources recommending a minimum sample of 20–30 participants per cluster [46] and others recommending using a ratio of participants to items of at least 10:1, suggesting a minimum sample of 50 participants [47]. Pertinent to the current study, other pain-related work has used samples based on 2^m^ where m = number of clustering variables [48,49]. We selected 5 variables a priori and, therefore, a minimum of 32 participants were required using this criterion. We acknowledge this is lower than other recommendations; therefore, as an exploratory study, we will focus on reporting effect sizes as future studies may aim to replicate these methods in a larger sample of individuals receiving rehabilitation.

### Identification of phenotypes

SPSS v. 25 (IBM, Armonk, NY) was used for all data analysis. PPT, somatization, depression, and anxiety subscales of the SCL-90R were Z-transformed and entered into a Principal Components Analysis with Varimax Rotation. The Principal Components Analysis was conducted prior to the hierarchical cluster analysis for the purpose of reducing dimensionality of the data. Components with eigenvalues greater than 1 were retained per Kaiser’s rule [50]. Saved regression factors were entered into a hierarchical cluster analysis with Ward’s clustering method and squared Euclidean distances. Cluster determination was based on the largest change in agglomeration coefficients between two adjacent steps.

Descriptive statistics were calculated for the total sample and derived clusters for all psychological, pain sensitivity, and outcome variables. A one-way ANOVA determined if derived subgroups significantly differed by somatization, depression, anxiety, and PPT. Next, either a Chi-Square Analysis for categorical variables or a one-way ANOVA for continuous variables determined differences in demographic, psychological (PCS, FABQ-PA, FABQ-W, TSK, PSEQ, PSQI), and clinical factors (pain duration) between the derived subgroups. Our a priori hypothesis was that individuals who reported higher levels of somatization, depression, and anxiety would also report higher levels of other negative pain-related psychological factors, such as catastrophizing. Cohen’s d effect sizes were calculated to determine the magnitude of difference between clusters for significant demographic and psychological variables with the following formula: (Mean of first cluster–Mean of second cluster/pooled standard deviation) and interpreted based on the following thresholds: 0.2 = small, 0.5 = medium, and 0.8 = large [51].

#### Differences in short-term clinical outcomes between phenotypes

Dependent variables were normally distributed (Kolmogorov-Smirnov/Shapiro-Wilk p>0.05). In two repeated measures ANOVAs, baseline cluster membership was included as the between subject factor and clinical pain intensity or ODI scores at baseline and four weeks as the within subject factor. A cluster x time interaction effect was examined with Bonferroni simple effects decomposition. Cohen’s d effect sizes were calculated to determine the magnitude of difference in short-term clinical outcomes of pain and disability within each derived subgroup. Cohen’s d was calculated with the following formula: (Mean of the Outcome at Baseline–Mean of the Outcome at 4-weeks/pooled standard deviation) and interpreted with the above effect size thresholds [51]. The p-value for significance was set at p<0.05 and did not adjust for multiple comparisons due the exploratory nature of this analysis.

## Results

Physical therapists had an average of 6.8 years of clinical practice, 62% were Orthopedic Clinical Specialists, and 100% reported they were completely aware of the American Physical Therapy Association’s Orthopedic Clinical Practice Guidelines for LBP and had a positive viewpoint. 100% reported collecting at least one measurement of psychological factors. The most provided interventions included: core stabilization, motor control exercises, education, and manual therapy.

### Identification and validation of three phenotypes

**Fig 1:**
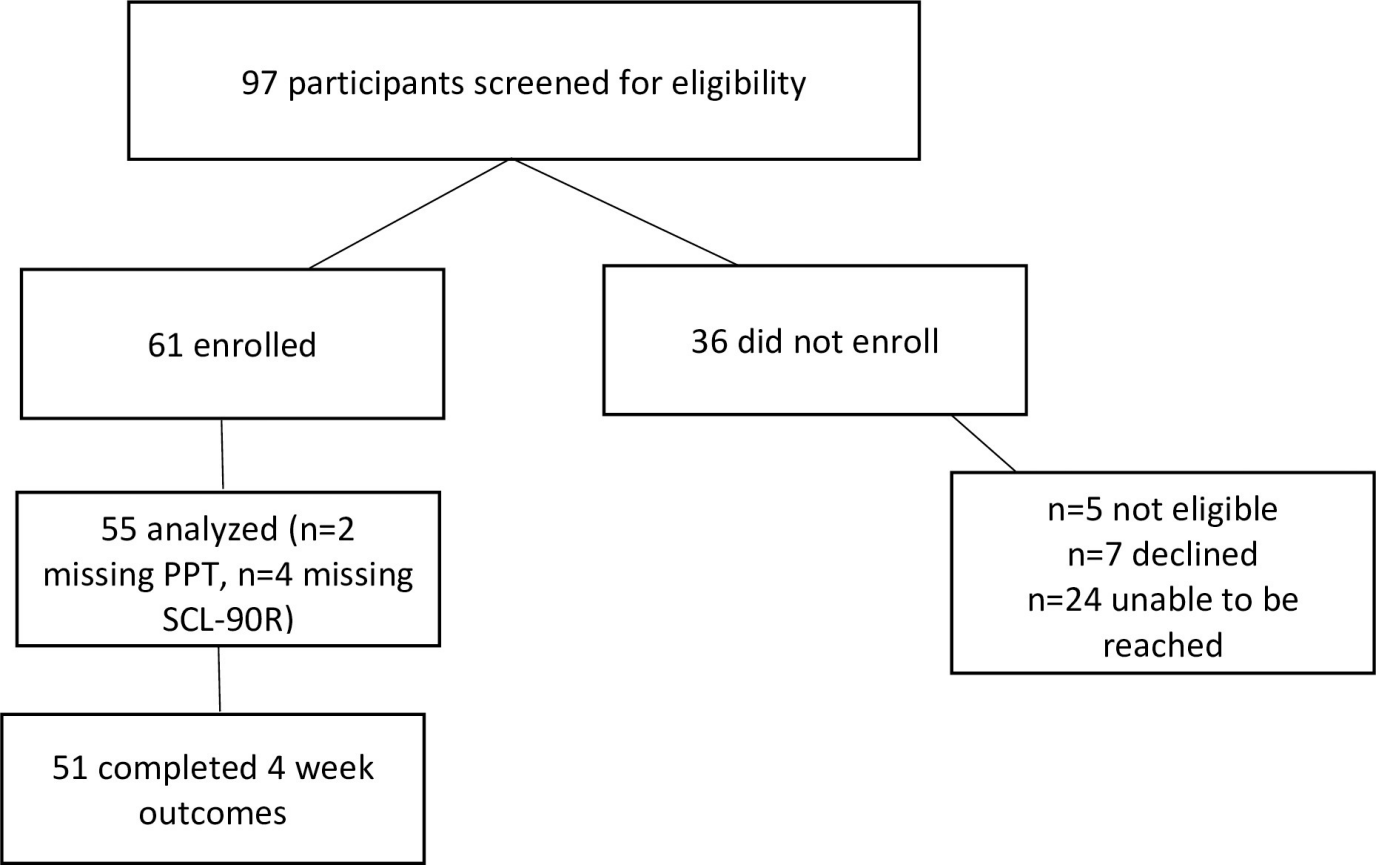
Flow chart of recruitment and enrollment.

Fifty-five participants completed all baseline outcomes with 90% follow-up rate at 4 weeks (**Fig 1**). Our sample size exceeded the recommended number of participants to conduct a cluster analysis [48,49]. Demographic and clinical data is presented in **Table 1.** Two principal components emerged with an eigenvalue greater than 1.0 [19,34] and a total variance explained = 89.01%. Component 1 consisted of somatization, depression, and anxiety. Component 2 consisted of PPT (**Table 2**). Factor loadings were sufficient without cross-loading.

**Table 1:** Demographic and clinical factors.

	Total Sample (n = 55) M or %	SD	Cluster 1 (n = 10) M or %	SD	Cluster 2 (n = 34) M or %		Cluster 3 (n = 11) M or %	SD	p-value between clusters
**Age** (years)	51.05	17.02	54.10	17.62	56.08	14.78	41.37	18.84	0.04*
Sex									0.20
Female	72.1		10.00		29.40		45.50		
Male	27.9		90.00		70.60		54.50		
**Ethnicity**									0.04*
Hispanic or Latino	4.9		20.00		0.00		9.10		
Not Hispanic or Latino	95.1		80.00		100.00		90.90		
**Race**									0.55
Caucasian	57.4		60.0		61.80		54.50		
African American	31.1		20.0		26.50		36.40		
Asian or Pacific Islander	4.9		0.00		2.90		0.00		
American Indian or Alaskan Native	1.6		0.00		2.90		0.00		
Other	4.9		20.00		2.90		0.00		
**Employment**									0.45
Full-Time Employed	39.3		30.00		38.20		27.30		
Part-Time Employed	13.1		0.00		14.70		27.30		
Unemployed	26.2		40.00		20.60		36.40		
Retired	21.3		30.00		26.50		9.10		
**Education Level**									0.83
Less than high school	14.8		30.00		14.70		9.10		
Graduated high school	4.9		0.00		5.90		9.10		
Some college	32.8		20.00		29.40		45.50		
Graduated from college	26.2		20.00		26.50		27.30		
Some post-graduate coursework	4.9		10.00		2.90		0.00		
Completed post graduate degree	16.4		20.00		20.60		9.10		
**Income**									0.24
Less than $20,000	26.7		33.30		23.50		27.30		
$20,000–35,000	13.3		33.30		2.90		27.30		
$35,000–50,000	15.0		11.10		11.80		18.20		
$50,000–70,000	18.3		0.00		26.50		18.20		
>$70,000	26.7		22.20		32.40		9.10		
**Back Pain Duration** (weeks)	135.51	252.83	237.20	480.44	127.06	208.90	75.82	91.84	0.36
**Previous episodes of back pain** (number)	55.89	155.79	26.90	36.42	52.59	115.52	118.55	307.42	0.39

M = mean, SD = standard deviation. p-value represents differences between clusters.

**Table 2:** Principal components analysis.

	Component 1	Component 2
Anxiety	0.89	
Depression	0.88	
Somatization	0.73	
PPT Upper Trapezius		0.94
PPT Lower Back		0.93

Kaiser-Meyer-Olkin measure of sampling adequacy = 0.62, Bartlett’s Test of Sphericity p<0.01.

**Fig 2:**
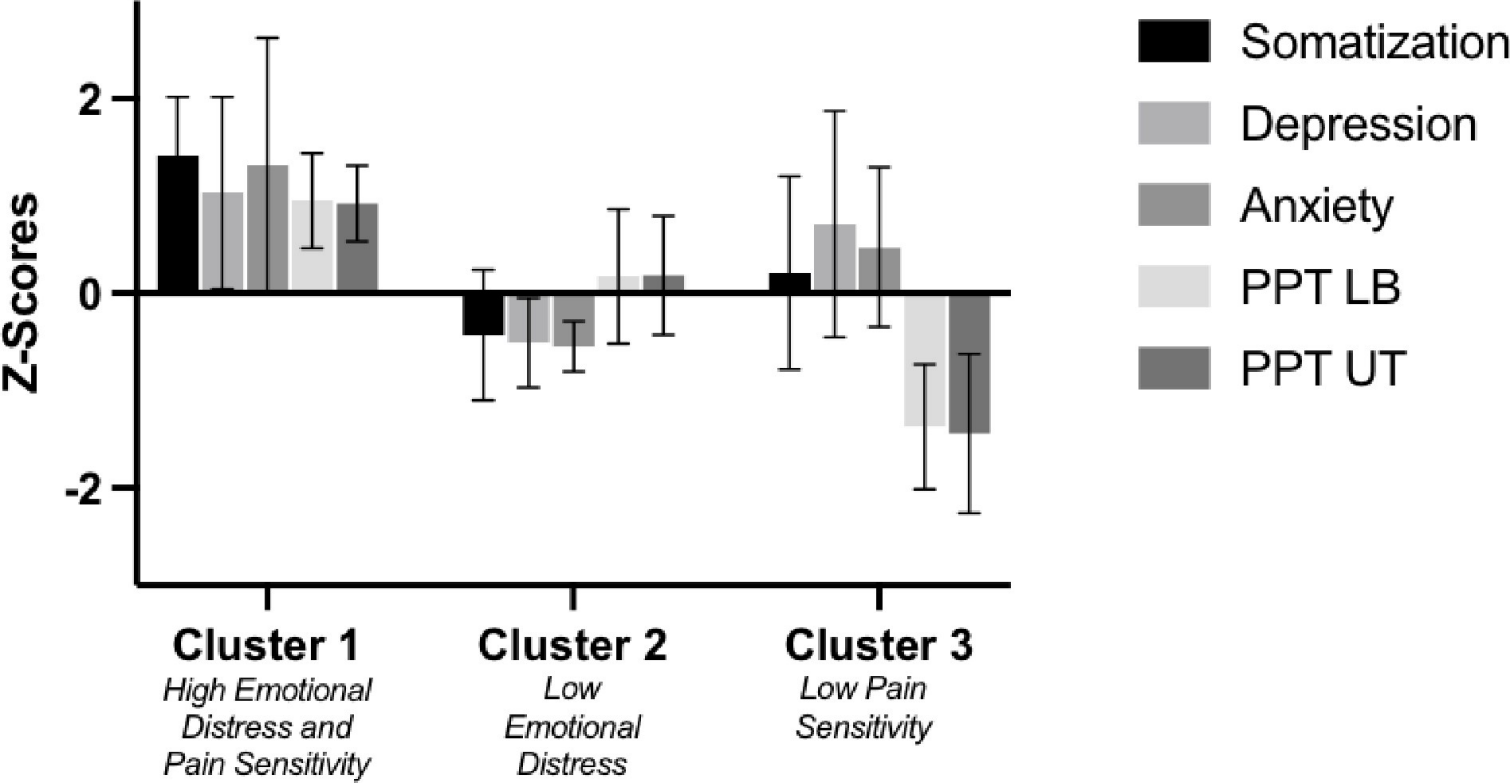
Z-scores of variables by phenotype.

Ward’s Hierarchical Cluster Analysis revealed 3 distinct clusters (48% change in agglomeration coefficient between adjacent steps) (**Fig 2**). Cluster 1 (n = 10) was the smallest and included individuals with high somatization, depression, anxiety, and pain sensitivity (“high emotional distress and pain sensitivity”). Cluster 2 was the largest (n = 34) and included individuals with low somatization, depression, and anxiety (“low emotional distress”). Cluster 2 was also characterized by average pain sensitivity. Cluster 3 (n = 11) was characterized by “low pain sensitivity.” This Cluster was also characterized by average distress. Somatization, depression, anxiety, and PPT significantly differed by cluster (p<0.01).

The numerical data is presented as standardized Z-score ± SD. Negative Z-scores reflect a lower somatization, depression, anxiety, and pain sensitivity. Positive Z-scores reflect higher somatization, depression, anxiety, and greater pain sensitivity.

Clusters significantly differed by age (p = 0.04) and ethnicity (p = 0.04) (**Table 1**). Individuals in the low pain sensitivity subgroup (Cluster 3) were younger than individuals in Cluster 1 (Cohen’s d = 0.69) and Cluster 2 (Cohen’s d = 0.87). In addition to having a low pain sensitivity, Cluster 3 was characterized by a younger age and being college educated. None of the individuals in the low emotional distress subgroup (Cluster 2) were Hispanic. Study site, duration of LBP, and previous number of episodes of LBP did not differ by phenotype (p>0.05).

As demonstrated in **Table 3**, Clusters significantly differed on all dimensions of the SCL-90R (p<0.05), PCS (p = 0.01), PSEQ (p = 0.03), and PSQI (p<0.01). In individuals with low emotional distress (Cluster 2), catastrophizing was significantly lower compared to Cluster 1 (Cohen’s d = 1.01) and Cluster 3 (Cohen’s d = 0.71) and self-efficacy was significantly higher compared to Cluster 1 (Cohen’s d = 0.96). Individuals with high emotional distress (Cluster 1) reported significantly poorer sleep patterns compared to Cluster 2 (Cohen’s d = 1.07). Clusters did not significantly differ by FABQ-PA (p = 0.67), FABQ-W (p = 0.15), nor TSK (p = 0.18).

**Table 3:** Total Scores of Pain-related psychological factors by cluster.

	Cluster 1 (n = 10) M	SD	Cluster 2 (n = 34) M	SD	Cluster 3 (n = 11) M	SD	p-value
SCL-90R							
Somatization	19.60	3.63	8.73	3.93	12.54	5.93	<0.01*
Obsessive-Compulsive	12.40	6.23	4.62	3.58	11.55	5.39	<0.01*
Interpersonal Sensitivity	5.80	4.41	1.76	1.77	5.55	5.42	0.01*
Depression	16.30	7.71	4.50	3.51	13.91	9.01	<0.01*
Anxiety	8.20	5.01	1.15	1.02	5.00	3.19	<0.01*
Hostility	2.80	2.39	1.09	1.33	2.55	3.55	0.04*
Phobic Anxiety	5.00	4.97	1.00	2.31	1.55	1.57	0.01*
Paranoid Ideation	4.00	5.16	0.82	1.59	4.37	4.45	0.02*
Psychoticism	3.40	3.17	.91	1.42	2.91	2.95	0.02*
PCS	22.70	12.93	11.00	10.18	18.90	11.99	0.01*
FABQ-PA	14.80	6.36	12.97	5.54	13.72	6.00	0.67
FABQ-W	8.10	11.11	8.51	11.21	15.81	10.81	0.15
TSK	27.00	6.25	23.00	6.77	25.72	5.73	0.18
PSEQ	30.30	17.67	45.27	13.00	40.91	16.93	0.03*
PSQI	14.40	5.62	8.82	4.38	10.37	3.79	0.01*

M = mean, SD = standard deviation, SCL-90R = Symptom Checklist 90 Revised, PCS = Pain Catastrophizing Scale, FABQ-PA = Fear-Avoidance Beliefs Questionnaire Physical Activity, FABQ-W = Fear-Avoidance Beliefs Questionnaire Work, TSK = Tampa Scale of Kinesiophobia, PSEQ = Pain Self-Efficacy Questionnaire, PSQI = Pittsburgh Sleep Quality Index, p-value represents differences between clusters at baseline. *indicates p-value <0.05.

### Short-term outcomes

**Fig 3:**
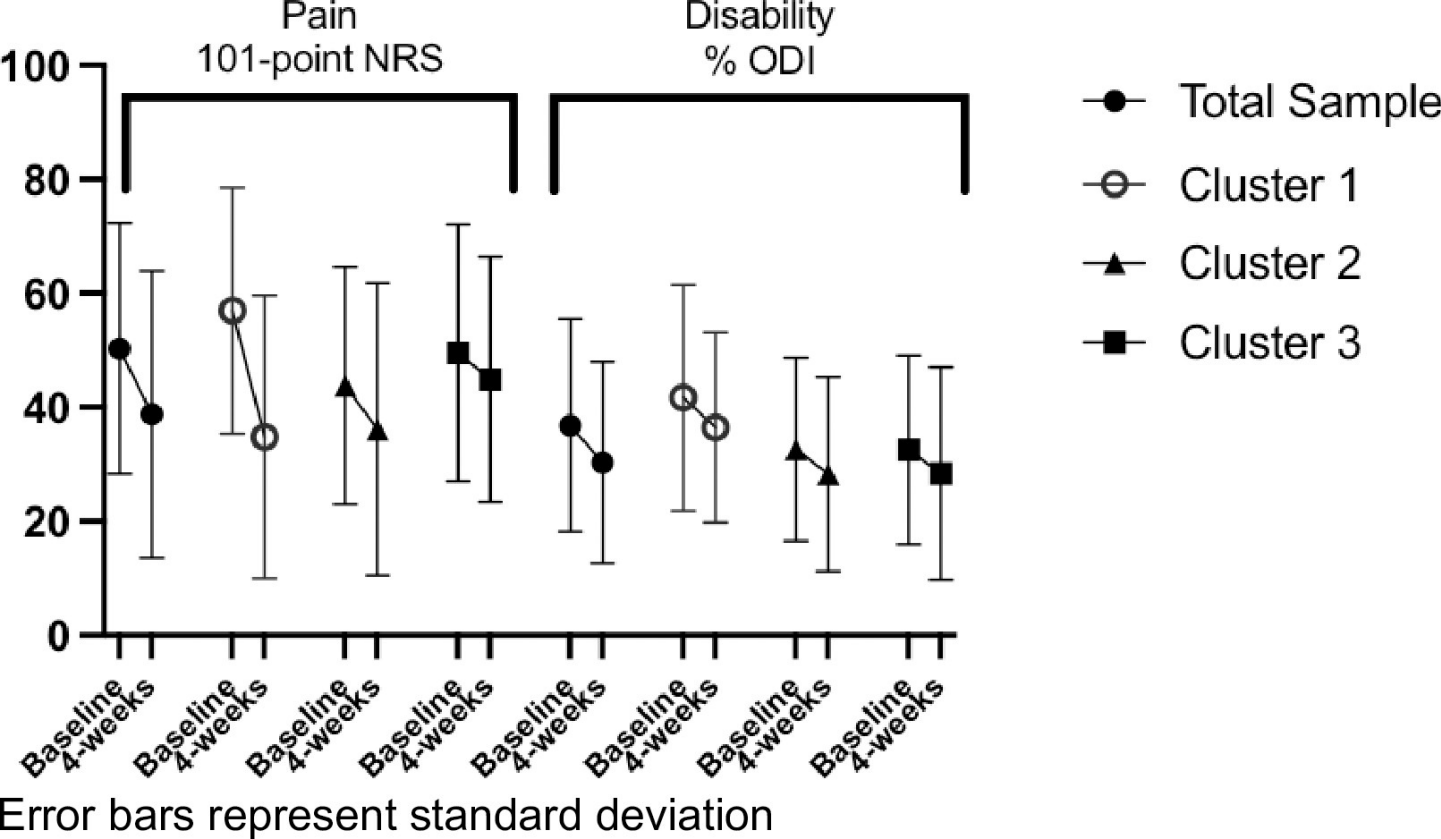
Changes in pain and disability ratings at baseline and 4-weeks for the total sample and by phenotype. Error bars represent standard deviation.

As demonstrated in **Fig 3**, a cluster x time interaction effect was not observed (F(2,47) = 2.52, p = 0.09, partial eta^2^ = 0.10) for pain ratings, suggesting pain ratings did not uniquely change by cluster after 4 weeks of physical therapy. A main effect of cluster membership (F(2,47) = 0.61, p = 0.55, partial eta^2^ = 0.03) was also not observed. A main effect of time (F(1,47) = 14.12, p<0.01, partial eta^2^ = 0.23) was observed, indicating pain ratings reduced after 4 weeks of physical therapy with a mean ± SE reduction in pain for the total sample = 11.52 ± 3.07. The cluster x time interaction effect did not reach our a priori threshold for significance (F(2,47) = 2.52, p = 0.09, partial eta^2^ = 0.10) for pain ratings due to the exploratory nature of this study.

Given the probability of chance was under 10%, we made a post-hoc decision to examine effect sizes to identify trends. Cluster 1 displayed a large effect size for reduction in pain ratings by 4 weeks (Cohen’s d = 0.95), Cluster 2 displayed a small to medium effect size (Cohen’s d = 0.32), and Cluster 3 displayed a small effect size (Cohen’s d = 0.21). These trends suggest individuals with high emotional distress and pain sensitivity (Cluster 1) have the largest reductions in pain in response to interventions provided by physical therapists.

For changes in disability, a cluster x time effect was not observed (F(2,47) = 0.03, p = 0.97, partial eta^2^ = 0.001). A main effect of cluster membership was not observed (F(2,47) = 1.07, p = 0.35, partial eta^2^ = 0.04) but a main effect of time was observed (F(1,47) = 6.57, p = 0.01, partial eta^2^ = 0.12) suggesting similar changes in disability over time. Results for disability and pain outcomes remained the same when controlling for age, ethnicity, study site, sex, and race. Given the probability of chance was under 10%, we made a post-hoc decision to examine effect sizes to identify trends. Cluster 1 displayed a small to medium effect size (Cohen’s d = 0.29), Cluster displayed a small to medium effect size (Cohen’s d = 0.26), and Cluster 3 displayed a small to medium effect size (Cohen’s d = 0.25). Collectively, the preliminary trends suggest small to medium reductions in disability across phenotypes.

## Discussion

We identified three clusters in patients with LBP using clinically relevant methods to collect psychological and pain sensitivity variables previously validated in a sample of individuals with varying pain conditions [16,17]. In our trial, three phenotypes were derived: 1) high emotional distress and pain sensitivity (n = 10), 2) low emotional distress (n = 34), 3) low pain sensitivity (n = 11).

Prior research [16,17] using the same clustering variables also identified three phenotypes. Our first phenotype paralleled the global symptoms cluster (higher pain sensitivity and psychological distress) [16,17] and our second phenotype paralleled the previously published adaptive cluster (lower pain sensitivity and psychological distress) [16,17]. In contrast, we identified a third phenotype characterized by individuals with low pain sensitivity (high PPT) while prior studies [16,17] identified a third phenotype characterized by high pain sensitivity (low PPT). These variations may be due to sample characteristics. An important difference between our work and that of others was that this trial was conducted in patients receiving rehabilitation for LBP while the others were conducted in healthy individuals and patients with temporomandibular dysfunction [16,17], individuals with complex persistent pain [17], and those seeking treatment at a multidisciplinary pain clinic [17]. Individuals with LBP receiving physical therapy may differ from individuals with complex persistent pain receiving care at a multidisciplinary pain clinic by key factors known to influence pain sensitivity, such as widespread chronic pain or activity level [52–54]. Furthermore, we acknowledge that this is an exploratory analysis that should be replicated in a larger sample size.

Phenotypes in the present study were further characterized by differences in age, ethnicity, and pain-related psychological factors. First, individuals demonstrating low pain sensitivity (Cluster 3) were significantly younger than the other clusters which is consistent with pain and aging literature demonstrating lower pain sensitivity in younger individuals compared to older adults [55–58]. Second, individuals in the high emotional distress and pain sensitivity phenotype (Cluster 1) had the largest proportion of individuals who were Hispanic. Consistent with our results, individuals who are Hispanic demonstrate elevated pain sensitivity in other studies [59]. Third, individuals in the high emotional distress and pain sensitivity phenotype (Cluster 1) also demonstrated elevated pain catastrophizing, lower pain-related self-efficacy, and poorer sleep patterns. Variability in psychological profiles exist in patients with LBP [12] and, consistent with previous literature, a subgroup of patients have higher depression, anxiety, pain catastrophizing [60], and poorer sleep quality [61].

We did not see significant interaction effects in our statistical models examining the clinical relevance of the phenotypes, possibly due to the sample size. However, exploratory analysis of effect sizes suggests these phenotypes may have different magnitudes of reduction in pain ratings after four weeks of physical therapy. A large effect size for reduction in pain was observed for individuals in the high emotional distress and pain sensitivity phenotype (Cluster 1). Thus, the phenotypes might include treatment moderators. Individuals with high fear and pain catastrophizing demonstrated the largest change in pain at 4 weeks when receiving physical therapy compared to those with low psychological factors [62]. While treatment was not prescribed across participating clinical sites in the current study, 100% of physical therapists in this study reported modifying treatment for those with elevated pain related psychological factors using psychologically informed principles [63–65], such as motivational interviewing and graded exposure. While speculative, this suggests physical therapists may be equipped, or at least knowledgeable, to successfully managing patients with elevated pain-related psychological factors. Although disability significantly reduced for the total sample, phenotypes did not differentiate short-term change in disability. While depression and somatization are predictors of disability in patients with LBP [66], improvement may vary according to baseline disability or STarT Back Tool Risk Category (which was not assessed in this study) and may help account for this result [67]. Future trials are needed in larger samples to validate the trends observed in this study and determine prognostic over treatment direction value of the phenotypes. Future research is needed to determine the prognostic capabilities of this subgroups. However, patients with high pain intensity at baseline are likely to be similar to other groups within a month. This may reflect a regression to the mean for this group; however, future studies would need to determine if this is occurring.

Nonetheless, this study advances phenotyping research. While previous studies have been conducted in experimental settings or using multiple questionnaires not feasible to administer in a clinical setting, this study took a clinically applicable approach based on published questionnaires/variables that were applied it to a novel setting, rehabilitation. We used one questionnaire, the SCL-90-R, that was collected online and could be efficiently applied in the clinical setting to form phenotypes. PPT data was quick to administer by a trained physical therapist during the patient’s appointment, providing ecological validity. Prospectively collecting data limits bias and is consistent with current phenotyping research priorities [11]. This protocol represents an advancement in implementing phenotyping research in the clinical setting.

There are limitations worth considering when interpreting the results of this study, specifically the sample size and lack of standardization of physical therapist interventions. As noted throughout this manuscript, we acknowledge that these findings are preliminary due to the small sample size. Furthermore, thresholds for classifying patients in each cluster based on the variable are unknown. Effect sizes might be influenced by the fairly small cluster samples sizes. Furthermore, as an observational study, this study lacks a comparator group. Future studies may aim to include a comparator group examining alternative methods of prognostic classification, such as the STaRT Back Tool. Although physical therapists were aware of Clinical Practice Guidelines (CPG) and reported routinely screening for negative pain-related psychological factors, it is unknown if clinicians were providing CPG informed treatment or interventions to target psychological factors. Additionally, inter-rater reliability of the PPT measurements were not collected. Data regarding interventions provided by physical therapists, frequency of treatment, and compliance may have provided greater context informing the lack of difference in short-term clinical outcomes.

The study represents an important first phase of future phenotyping trials conducted in the rehabilitation setting. Future trials with larger sample sizes are needed to validate the trends reported in the results for short-term pain and disability outcomes. Next, identifying key intervention targets related phenotype membership and matching interventions to these targets is an essential next step to validate and implement these approaches into clinical care.

## Conclusion

Similar to previously published studies, three exploratory phenotypes in a small sample of patients receiving physical therapy for LBP were identified when clustering based on somatization, depression, anxiety, and PPT characteristics. Similar phenotypes were observed in prior studies [16,17] with the exception of the phenotype based on pain sensitivity. As an exploratory analysis, we also investigated the relevance of phenotypes to short-term clinical outcomes of pain and disability. Effect sizes for reductions in pain suggest different magnitudes of change by phenotype. Specifically, a large effect size was observed for reductions in pain for individuals with high emotional distress and pain sensitivity. While future research is needed to validate these results in a larger sample, the clinically applicable design provides a novel approach for future clinical trials and suggests phenotyping is feasible in clinical practice.

## Supporting information

S1 File (XLSX)
